# Effect of Preheating Temperature on Geometry and Mechanical Properties of Laser Cladding-Based Stellite 6/WC Coating

**DOI:** 10.3390/ma15113952

**Published:** 2022-06-01

**Authors:** Teng Wu, Wenqing Shi, Linyi Xie, Meimei Gong, Jiang Huang, Yuping Xie, Kuanfang He

**Affiliations:** 1 School of Electronics and Information Engineering, Guangdong Ocean University, Zhanjiang 524088, China; wuteng97921@163.com (T.W.); gaxly19980104@163.com (L.X.); gmeimei1124@163.com (M.G.); huangjiang@gdou.edu.cn (J.H.); xiexie160703@163.com (Y.X.); 2 School of Mechatronic Engineering and Automation, Foshan University, Foshan 528000, China; hkf791113@163.com

**Keywords:** laser cladding, composite coating, microstructure, hardness, wear property

## Abstract

The effect of 60Si2Mn substrate preheating on the forming quality and mechanical properties of cobalt-based tungsten carbide composite coating was investigated. Substrate preheating was divided into four classes (room temperature, 150 °C, 250 °C, and 350 °C). The morphology, microstructure, and distribution of elements of the coating were analyzed using a two-color laser handheld 3D scanner, a scanning electron microscope (SEM), and an energy dispersive X-ray spectrometer (EDX), respectively. The hardness and wear properties of the cladding layer were characterized through a microhardness tester and a friction wear experiment. The research results show that the substrate preheating temperature is directly proportional to the height of the composite coating. The solidification characteristics of the Stellite 6/WC cladding layer structure are not obviously changed at substrate preheating temperatures of room temperature, 150 °C, and 250 °C. The solidified structure is even more complex at a substrate preheating temperature of 350 °C. At this moment, the microstructure of the cladding layer is mainly various blocky, petaloid, and flower-like precipitates. The hardness and wear properties of the cladding layer are optimal at a substrate preheating temperature of 350 °C in terms of mechanical properties.

## 1. Introduction

60Si2Mn silicomanganese spring steel (density: 7.85 g/cm^3^) has moderate strength and a low price and it is widely applied in the manufacturing of rolling stock weight-bearing springs, agrimotor rotary blades, etc. However, its application is restricted under severe conditions because of the low hardness and poor friction wear property. Laser cladding (LC) technology is considered one of the effective methods to prepare wear-resisting coating and mitigate the wear-out failure of the part surface. Owing to such advantages as high-energy density, small workpiece deformation, and strong capability for metallurgical bonding to the substrate, this technology has attracted the attention from many research scholars [1,2,3,4]. To meet the increasing performance requirements for cladding materials, introducing metal-ceramic composite coatings, such as Fe-based alloy + WC composite coating [5,6], Ni-based alloy + TiC composite coating [7,8], and cobalt-based alloy + WC composite coating, into the LC process to effectively strengthen the microhardness and wear properties of the part surface has gradually become one of the research emphases [9,10] in recent years because it is difficult for any single-phase coating to meet the use requirements. However, many problems still exist in repair and reinforcement applications, and the forming quality of coatings is one of the importance factors confining the development of LC [11].

Quality defects, such as unmelted powder, micro-cracks, and pores, can be easily caused by physical property differences between materials, the unreasonable selection of process parameters, etc., in the LC process [12,13]. To enhance the forming quality of the cladding layer, domestic and overseas scholars reported the relationship between the coating’s forming quality and LC process parameters [14,15] in the preparation process of composite coatings. Some researchers also reinforced the comprehensive performance of coatings using such methods as changing the laser cladding spot [16,17,18] and changing the processing path [19]. Although there are many ways to reduce coatings defects, the results are not very effective. For the past few years, with further study into the LC process, researchers have found that the combination of the assisting laser processing process with laser cladding can well enhance the forming quality of the composite coating. Qi et al. [20] used the magnetic-field-assisted LC to create a TiB2/metal composite material on a titanium alloy’s substrate. They believed that the magnetic field could have a mixing effect on the molten pool, thus refining the TiB2/metal composite coatings. The TiB2 grain size decreases as the magnetic field intensity increases, and the coating defects reduce. The assisting laser processing process can also improve the wear and corrosion resistance properties of the coating. Wen et al. [21] successfully prepared a crack-free FeCrCoAlMn_0.5_Mo_0.1_ coating using ultrasound-assisted LC technology. The research results showed that both wear and corrosion resistance properties of the coating are better than those of the substrate. Liu et al. [22] prepared a Ni60CuMoW coating on the surface of medium carbon steel 45 using a mechanical-vibration-assisted LC composite surface modification process. They believed that mechanical vibration can apparently strengthen the corrosion and wear properties of the cladding layer. According to Reference [23], its authors conducted research on a nickel-based WC composite material by introducing an assisting process—preheating. This research showed that preheating the substrate with a heater can reduce the temperature gradient (G), the solidification rate (R), and the cracking susceptibility of the cladding layer. Farahmand et al. [24] confirmed that the defects of the cladding layer are reduced with the assistance of preheating. Bidron et al. [25] attempted to reduce crack defects in the cladding layer by using induction preheating for a CM-247LC high-temperature alloy, and the results of the study showed that a cladding layer without crack defects could be obtained when the substrate preheating temperature was 1050 °C. To further verify the effects of various parameters in the substrate preheating-assisted process on the crack density of the cladding layer, Soffel et al. [26] used direct metal deposition to prepare nickel-based coatings on the surface of stainless steel and investigated the effects of substrate temperature, specimen geometry, deposition parameters, and scanning strategy on the crack density, and the results showed that increasing the substrate temperature and reducing the specimen size by laser preheating could obtain a crack-free deposited structure. Liu et al. [27] used a combination of numerical simulations and experiments to verify that the cooling rate decreases with increasing heat flow density of the preheating heat source, and the temperature gradient decreases rapidly with increasing distance from the surface

However, the existing research literature mainly discusses the effects of the substrate preheating auxiliary process parameters on the geometrical morphology of the cladding layer, and there are limited articles reporting on the effects of substrate preheating temperature on the mechanical properties of the cladding layer. Therefore, in this paper, cobalt-based tungsten carbide composite coating was prepared on the 60Si2Mn surface using a combination process of LC and substrate preheating, and the effect of substrate preheating on the morphology, structure, distribution of elements, and performance of the cobalt-based tungsten carbide composite coating was investigated.

## 2. Research Methods

### 2.1. Materials

**Figure 1:**
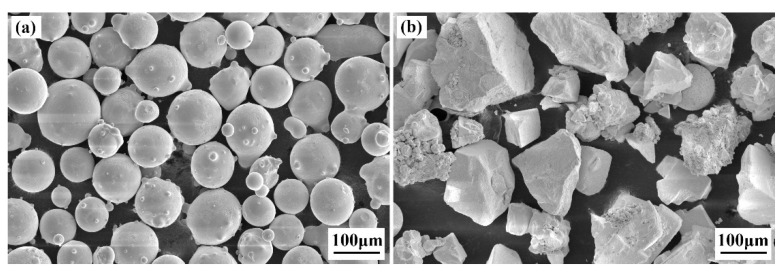
SEM morphology of the cladding material: (**a**) Stellite 6 powder; (**b**) WC powder.

60Si2Mn steel was used as a substrate material. The specimen dimensions were 40 mm (Length) × 20 mm (Width) × 2 mm (Thickness). Table 1 shows its chemical composition. Before LC, the steel substrate was ground first and then cleaned with alcohol to remove the oxide layer. Laser surface modification was performed on 60Si2Mn steel by the combination method of LC and substrate preheating. The cladding layer material consisted of Stellite 6 and WC powders (purity: 99.8%). Figure 1 shows the SEM morphology of the cladding material. The chemical composition of Stellite 6 powders is shown in Table 2. The Stellite 6 spherical powders and the added WC blocky powders have average grain sizes of about 30–150 μm and 65–245 μm, respectively. For the composite coating, we finally selected a weight ratio of 7:3 for the mixing of Stellite 6 and WC, according to the crack susceptibility of the Stellite 6/WC metal-based composite material with different WC contents reported in References [28,29,30,31]. The Stellite 6 and WC powders were mixed in a v-mixer at a speed of 29 r/min for 2 h.

### 2.2. Laser Cladding Process Parameters

**Figure 2:**
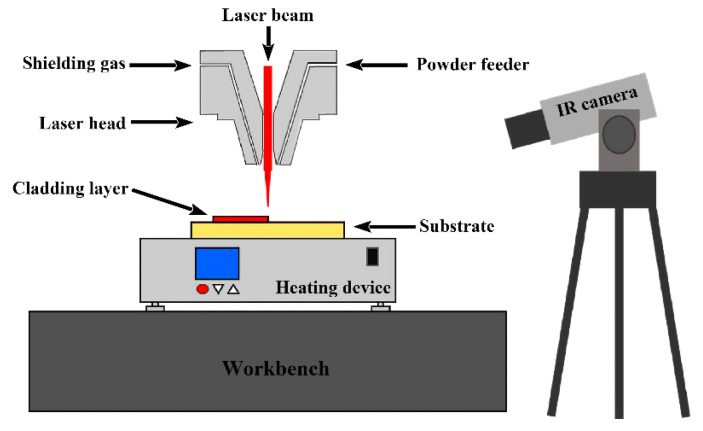
Schematic diagram of LC process.

**Figure 3:**
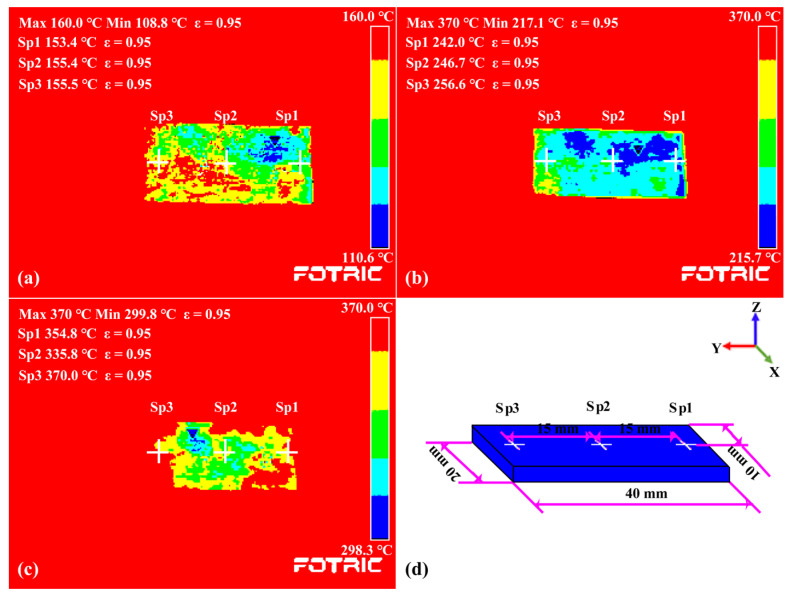
Temperature contour diagrams when the substrate was preheated to a predetermined temperature: (**a**) 150 °C; (**b**) 250 °C; (**c**) 350 °C; (**d**) temperature monitoring points.

Figure 2 depicts a schematic representation of the LC process. The laser additive manufacturing process was controlled using a 2 kW continuous fiber laser processing system (laser source model: MFSC 2000 W, manufacturer: Maxphotonics Co., Ltd., Shenzhen, China) and a synchronous powder feeding system (model: HW-05SF, manufacturer: Dongguan City HW Laser Equipment Co., Ltd., Dongguan, China). The laser beam wavelength was 1080 ± 5 nm, the beam parameter product of the laser source is 0.5–2.5 mm × mrad, and the spot diameters of the laser beam acting on the surface of the substrate were 2.10 mm. The substrate was preheated on a constant-temperature heating platform (model: DB-XAB, working voltage: 220 V, maximum operating temperature: 400 °C, manufacturer: Shanghai LICHEN-BX instrument technology CO., Ltd., Shanghai, China). The preheating temperature was divided into four classes: room temperature, 150 °C, 250 °C, and 350 °C. The entire heating process was monitored by a thermal imager (model: 225s, manufacturer: FOTRIC INC., Shanghai, China). Figure 3 shows temperature contour diagrams when the substrate was preheated to a predetermined temperature, where SP1 (10, 5, 2), SP2 (10, 20, 2), and SP3 (10, 35, 2) were temperature monitoring points. The preparation process parameters of the metal-based composite coating are shown in Table 3. According to the reference [14] and the trial-and-error results before the experiment, the laser power for this experiment is 1500 W, the scanning speed is 5 mm/s, and the feeding speed is 13 g/min. Both the carrier gas and shielding gas were argon (with a gas flow rate of 10 L/min) in the LC process. The distance between the laser cladding head and the substrate was 20 mm.

### 2.3. Surface Conditions and Microstructure Characterization

After LC was completed, the morphology of single-pass coating was characterized with a two-color laser handheld 3D scanner (model: Prince335, scanning accuracy: 0.03 mm, manufacturer: Hangzhou Scantech Co., Ltd., Hangzhou, China). The cladding specimens were transversely cut with an EDM CNC wire cutting machine (model: QC350K, manufacturer: Sichuan Shenyang CNC Machinery Co., Ltd., Chengdu, China), ground with metallographic sandpaper with a grain size of 180–3000, and polished with 1 µm diamond paste. To reveal the microstructure of the cross-sectional coating of the prepared specimens, the polished specimens were etched with a mixture of concentrated nitric acid (HNO_3_) and hydrochloric acid (HCI) with a molar ratio of 1:3, i.e., so-called nitrohydrochloric acid. Macromorphology, microstructure, and qualitative elemental analyses of the coating were performed with SEM (model: JSM 6460, manufacturer: JEOL Ltd., Tokyo, Japan) and EDX (EDAX-GENESIS) devices. The included cladding angle (α) between the coatings and the substrate was measured using Image J software (version number: 1.8.0, manufacturer: National Institutes of Health, Bethesda, MD, USA) so as to evaluate the forming quality of the composite coating. The phase composition of the Stellite 6/WC composite coating was obtained using XRD (model: D/max2550VL/PC, manufacturer: Rigaku Corporation, Tokyo, Japan), with the radiation source being Cu Kα.

### 2.4. Mechanical Property Test

**Figure 4:**
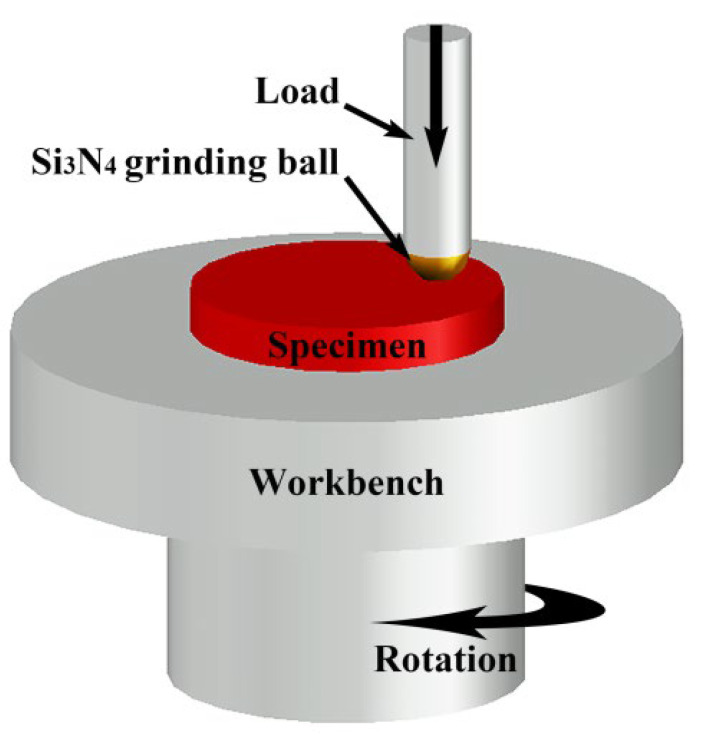
Schematic diagram of friction wear test.

For mechanical properties, the microhardness of the metal-based Stellite 6/WC coating was measured along the cladding depth (radial direction) with a Vickers microhardness tester (model: WILSON VH1202, manufacturer: BUEHLER, Lake Bluff, IL, USA) under the conditions of 100 g load and 10 s loading time. A wear resistance property test was performed on the cladding specimens with a friction wear tester (model: CFT-I, manufacturer: Lanzhou Zhongke Kaihua Technology Co., Ltd., Lanzhou, China) at room temperature. Figure 4 shows a schematic representation of the wear test. The friction wear test was conducted under dry sliding conditions. The grinding material was a φ4 mm silicon nitride ceramic (Si_3_N_4_) ball. The mode of motion was rotary motion. The applied load was 40 N. The rotational speed was 200 revolution per minute (RPM). The rotation radius was 0.8 mm. The frequency was 1 Hz. The test time for each specimen was 30 min. A high-precision analytical balance (model: FA2104N, accuracy: 0.1 mg, manufacturer: Shanghai Precision Scientific Instrument Co., Ltd., Shanghai, China) was used to weigh the specimens before and after the test. To calculate the mass loss, each specimen was weighed five times (averaged). The mass loss was converted into a volume loss by being divided by the corresponding density value. The weighted density method was used to transform the mass loss in the composite material’s cladding layer into a volume loss [32]. Microstructure analysis of the worn specimens was performed with an SEM. It should be noted that the microstructural characteristics, microhardness, and wear resistance tests of the cladding layers in this study were all conducted at the same cross-section.

## 3. Results and Discussion

### 3.1. Surface Morphology of Different Specimens

**Figure 5:**
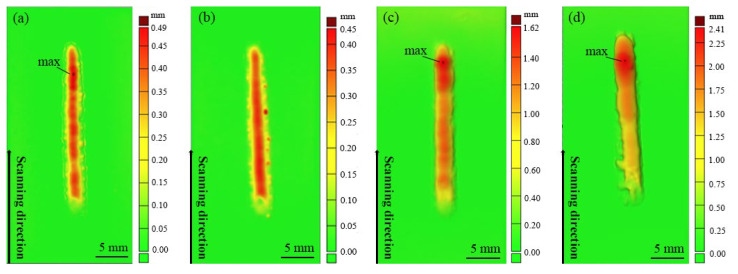
Three-dimensional laser-scanned morphology of the Stellite 6/WC cladding layer: (**a**) S1; (**b**) S2; (**c**) S3; (**d**) S4.

Figure 5 shows the 3D scanning morphology of the cladding layer when the substrate is heated to different temperatures. It can be seen that the morphology of each specimen is significantly different from that of others. As shown in Figure 5a,b, when the substrate is not preheated and when it is preheated to 150 °C, the height of the metal-based composite coating basically remains consistent, but the surface height differences are significantly different. Substrate preheating contributes to a reduction in the surface height difference of the coating because substrate preheating increases the duration of the molten pool, and the cladding layer in the molten state tends to level out under the combined action of coating surface tension and gravity. Figure 5c,d show the surface morphologies of the composite coating when the substrate is heated to 250 °C and 350 °C, respectively. The height of the Stellite 6/WC cladding layer is found to increase, as the substrate preheating temperature rises. The reasons for this phenomenon are summarized as follows: (1) In the event that other factors are unchanged, the increase in the preheating temperature in the LC process causes an increase in thermal output received by the substrate and the cladding powder, and thus an increase in molten powder volume. The molten substance diffuses on the substrate under gravity to form a high cladding layer. (2) It may be related to the characteristic of slow heat release when heat is accumulated in the WC powder, causing more heat accumulation in the coatings and an increase in the composite coating height. In addition, applying an excessive preheating temperature to the substrate may cause an increase in the surface height difference of the coating because heat accumulation appears in the coating in the LC process, and the heat at the endpoint of cladding is higher than that at the start point so that the maximum cladding height appears at the endpoint, forming a large height difference.

### 3.2. Cross-Sectional Characteristics of Different Specimens

**Figure 6:**
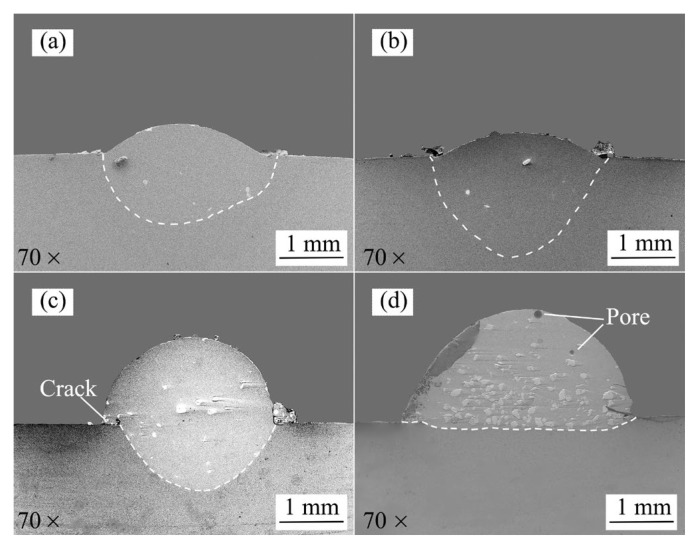
Cross-sectional morphologies of the Stellite 6/WC cladding layer: (**a**) S1; (**b**) S2; (**c**) S3; (**d**) S4.

The cross-sectional morphologies of the different specimens are given in Figure 6. As shown in Figure 6a,b, when the substrate preheating temperature is low, the forming effects of the Stellite 6/WC cladding layer are good. When the substrate preheating temperature is 250 °C, as shown in Figure 6c, the crack defect appears at the junction of the coating and the substrate. This is because the temperature gradient at the junction of the cladding layer and the substrate is large, and when the laser heat source moves along the scanning direction, the shrinkage of the coating along the scanning direction is constrained by the substrate, which makes the coating subject to tensile stress [33]. In addition, during the cooling of the coating, the high heat transfer of the metal makes some of the heat in the coating pass through the cladding layer into the substrate for release, resulting in high tensile stresses on the coating, which eventually lead to cracks at the junction between the molten coating and the substrate. As the substrate preheating temperature was further increased, porous defects appeared in the molten coating when the substrate preheating temperature was 350 °C (see Figure 6c). This phenomenon may be due to the fact that with the increase in the substrate preheating temperature, although the melt pool exists for a longer time, it also prompts a substantial increase in the height of the cladding layer so that the time required for the gas and slag in the melt pool to rise to the surface of the cladding layer exceeds the time when the melt pool is in a liquid state, and the gas and slag do not have time to fully overflow in the bottom layer, forming porous defects. In addition, some unmelted WC particles are distributed in the cladding layer and molten pool of each specimen, which was caused by the high melting point of WC (2870 ± 50). To sum up, it can be concluded that applying an excessive preheating temperature to the substrate may cause defects in the Stellite 6/WC cladding layer.

**Figure 7:**
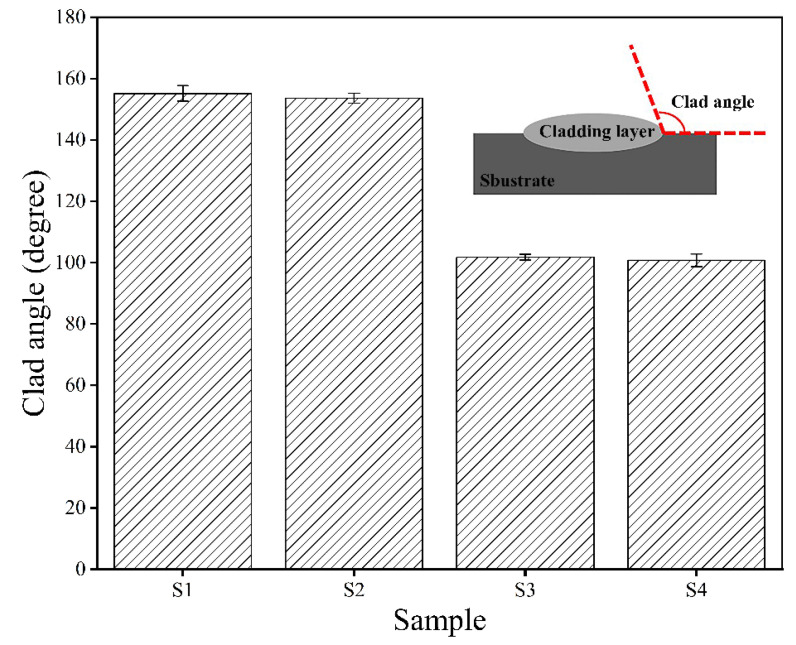
Cladding angles of Stellite 6/WC cladding layer of different specimens.

To better understand the relationship between preheating temperature and forming quality of the cladding layer, we introduced a cladding angle between the substrate and the composite coating. It is closely related to the height and width of the cladding layer and is an important basis to determine whether a metallurgical bonding is formed. It has been reported in Reference [34] that the larger the cladding angle value is, the fuller the spread of the cladding material on the substrate surface will be. The influence of the substrate preheating temperature on the cladding angle is seen in Figure 7. The results reveal that when the substrate preheating temperature rises, the cladding angle decreases from 155.16 degrees to 100.74 degrees. The reason for this phenomenon is that with the rise in the preheating temperature, the thermal input received by the substrate and the cladding powder rises; so much energy is used to melt the powder and the substrate, causing an increase in the coating height and a decrease in the cladding angle.

### 3.3. Microanalysis of Different Specimens

**Figure 8:**
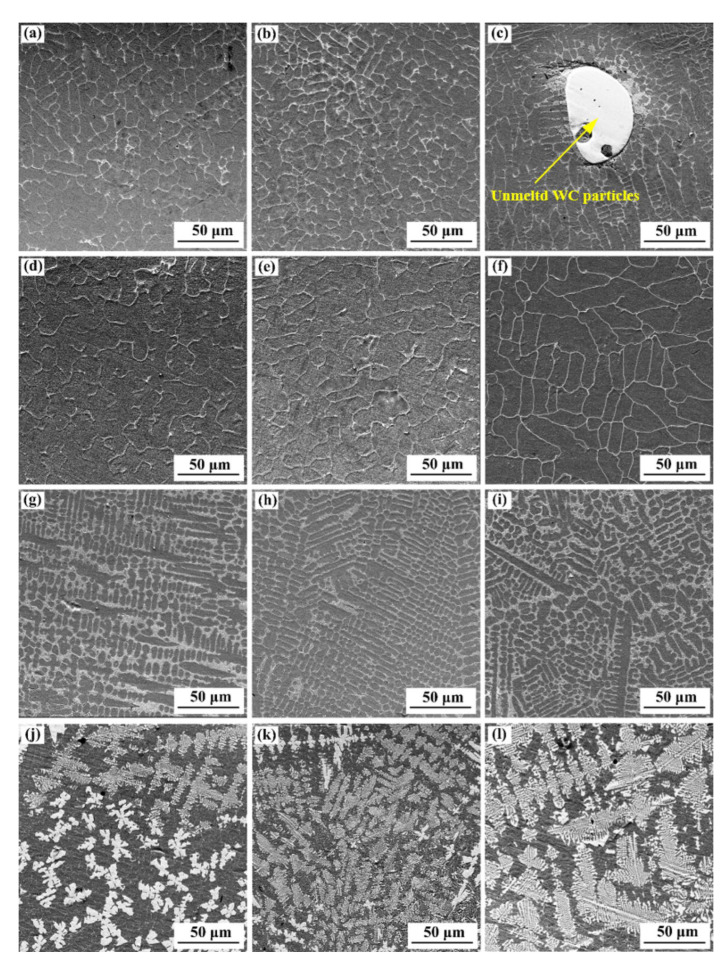
SEM morphologies of different specimens: (**a**) top part of S1; (**b**) middle part of S1; (**c**) bottom part of S1; (**d**) top part of S2; (**e**) middle part of S2; (**f**) bottom part of S2; (**g**) top part of S3; (**h**) middle part of S3; (**i**) bottom part of S3; (**j**) top part of S4; (**k**) middle part of S4; (**l**) bottom part of S4.

The microstructure of the cladding layer of different specimens is given in Figure 8. As shown in Figure 8a,d,g, a considerable amount of isometric crystals are formed in the top part of the composite coating because the heating and heat dissipation in the top part of the composite coating are uniform so that the growth rates of crystal nucleus are basically the same in different directions. Figure 8b,e,h show the SEM images of the middle part of the cladding layer of different specimens. Considerable amounts of isometric crystals are formed in the middle of the coatings. The reason for this phenomenon is that the R of the crystal nucleus is basically consistent in different directions because of the long distance between the middle part of the coatings and the substrate and relatively small G. As shown in Figure 8c,f,i, considerable amounts of cellular or columnar crystals are formed in the bottom part of the composite coating. Since the distance between the bottom of the coatings and the substrate is small and the G is large, the above-mentioned phenomenon is caused in the bottom part of the coating. To sum up, the solidification characteristics of the Stellite 6/WC cladding layer structure are not obviously changed at substrate preheating temperatures of room temperature, 150 °C, and 250 °C, but the solidification behavior of the interdendritic eutectic (grayish black) structure is affected and the volume of eutectics (grayish white) increases with the rise in the substrate preheating temperature.

Figure 8j–l show the SEM morphologies of the top, middle, and bottom parts of the composite coating when the substrate is preheated to 350 °C. It can be seen that the solidification process and microstructure in the cladding layer are obviously different from the solidification characteristics of the cladding layer of the other three specimens and the solidification structure is more complex. At this moment, the microstructure of the cladding layer is mainly various blocky, petaloid, and flower-like precipitates. This phenomenon has been also reported in Reference [35]. The reason is that with the rise in the substrate preheating temperature, WC with large particles may be locally molten while WC with small particles is completely molten into the Stellite melt in the LC process due to the high melting point of tungsten carbide [36]. After the cooling and solidification process starts, locally molten WC becomes a heterogeneous nucleation core of complex precipitates, and various blocky and petaloid precipitates grow according to the current cooling rate and temperature gradient. The reason for forming flower-like precipitate is that WC completely dissolved in the molten pool is re-solidified and precipitated according to the temperature gradient and heat flow direction in the solidification process.

**Figure 9:**
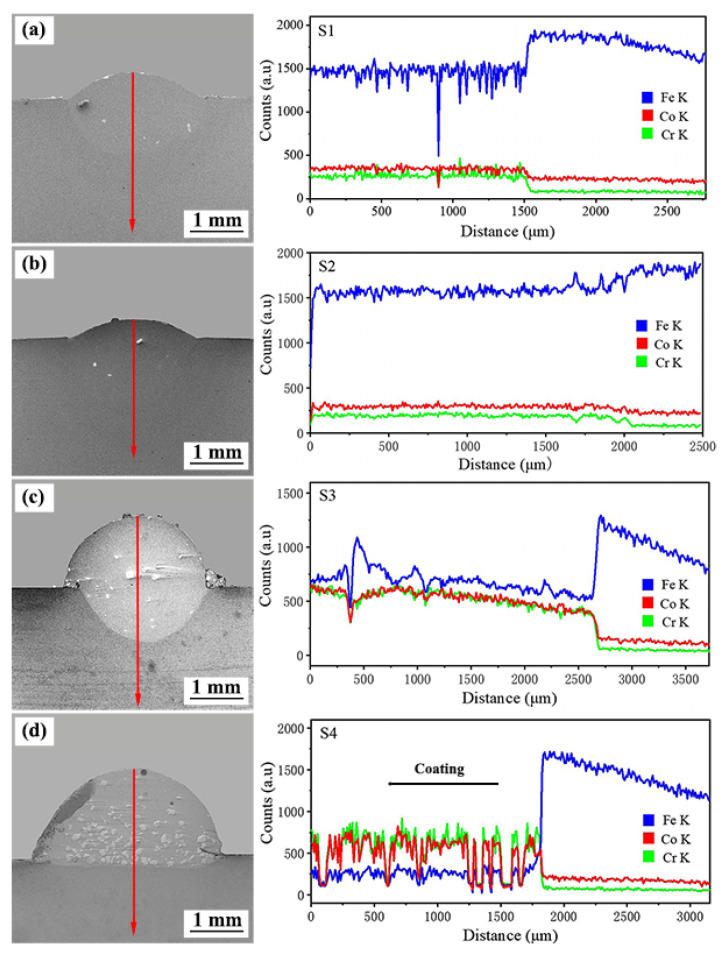
Line EDX between the Stellite 6/WC composite coating and the substrate of different specimens: (**a**) S1; (**b**) S2; (**c**) S3; (**d**) S4.

The distribution of elements in the coating and interface regions was analyzed using EDX line scanning in the direction perpendicular to the interface fusion line, as shown in Figure 9. By referring to the chemical composition of the substrate and the powder, it can be seen that the content of the Fe element in the substrate is the highest. As the testing location is deepened, the Fe element molten on the substrate surface enters the coating. The content of the Fe element is on the rise from the composite coating to the substrate. This is contrary to the Co and Cr elements, indicating that interdiffusion exists between the coating and the substrate, dilution occurs, and metallurgical bonding is achieved between the coating and the substrate. In addition, the dilution phenomenon in the Stellite 6/WC cladding layer is mitigated with the raising of the preheating temperature of the substrate. The reason for this might be that the height of the composite coating increases with the rises in the substrate preheating temperature so that elements in the substrate cannot fully rise to the cladding layer, and the difference in the content of the Fe element between the substrate and the composite coating increases.

### 3.4. Microhardness Distribution of Different Specimens

**Figure 10:**
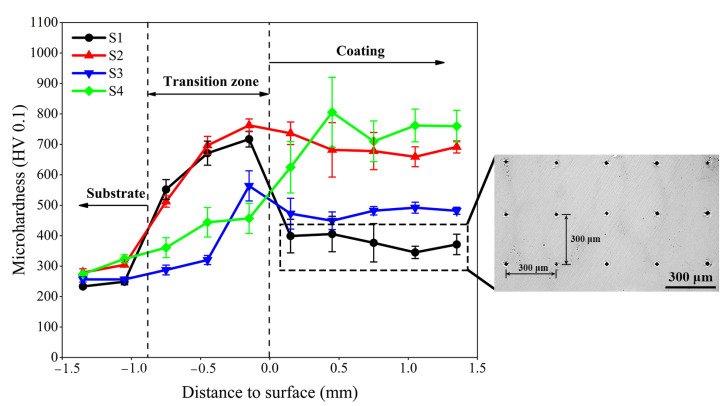
Microhardness distribution along the cross-sectional depth of the coating.

**Figure 11:**
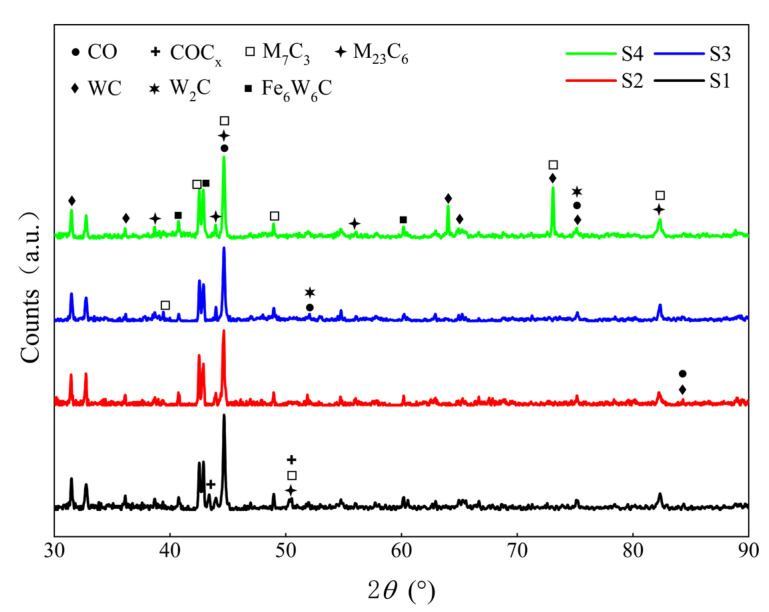
XRD chromatogram of the Stellite 6/WC composite coating of different specimens.

The microhardness distribution results of different specimens along the cross-sectional depth are given in Figure 10. As indicated in the graph, the average microhardness values of the Stellite 6/WC composite coating of several specimens are in the following order from high to low: S4 (732.38 HV_0.1_) > S2 (689.37 HV_0.1_) > S3 (475.51 HV_0.1_) > S1 (379.50 HV_0.1_). Based on the results of Farahmand et al. [24], they concluded that if the coating received too much heat input it would lead to WC dissolution and lower coating hardness. When the substrate is preheated at 150 °C and 250 °C, the hardness of the clad layer is consistent with the above findings. However, the hardness of the cladding layer of the S4 specimen is the highest when the substrate was preheated at 350 °C, about 2.45 times that of the substrate 60Si2Mn. Analysis of the XRD test results of the cladding layer of different specimens in Figure 11 shows that more carbide M_7_C3 and WC hard phases are formed in the cladding layer of the S4 specimen than in other specimens. In addition, the dilution rate of the S4 cladding layer is lower. Therefore, the microhardness of the S2 clad layer is between S3 and S4. It has been also reported in Reference [37] that the lower dilution rate resulted in higher microhardness of the composite coating. For some specimens (S1, S2, and S3), the microhardness in the cladding layer zone is smaller than that in the transition zone. This is mainly caused by the settling process after the crystallization of large WC particles.

### 3.5. Wear Property Analysis

**Figure 12:**
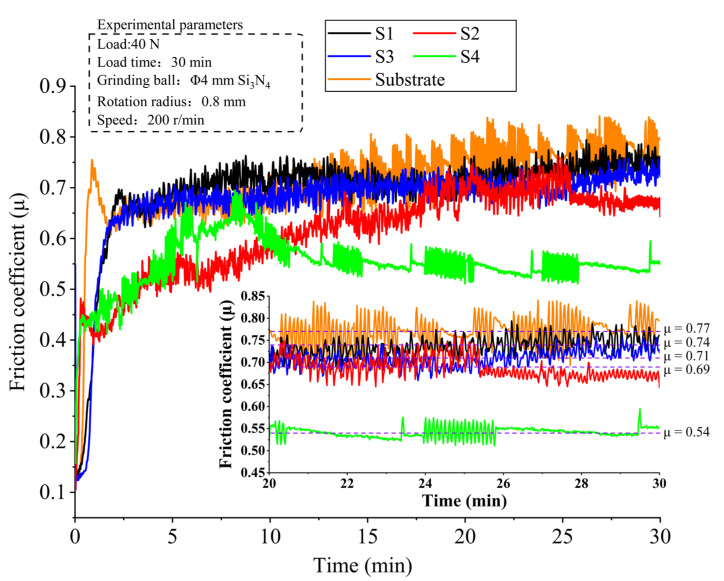
Friction coefficient curves of the substrate and the Stellite 6/WC composite coatings of different specimens.

**Figure 13:**
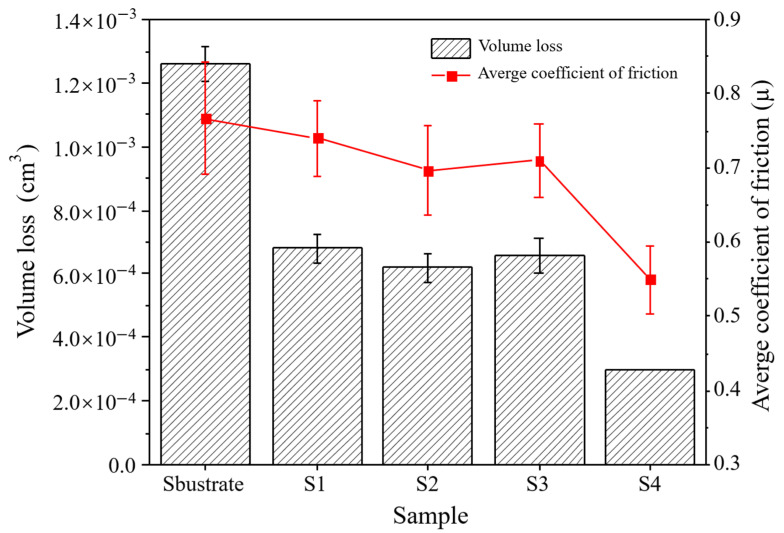
Wear volumes and average friction coefficients of the Stellite 6/WC composite coatings of different specimens.

One of the most significant property characterization methods for composite coatings is wear property. As a result, the friction coefficient, wear volume, and wear profile were used to assess each specimen’s wear property. In order to objectively reflect each specimen’s wear property, each specimen was tested under identical test settings. Figure 12 shows the friction coefficient vs. time graphs after dry friction wear of the 60Si2Mn substrate and the Stellite 6/WC cladding layer of different specimens. The results show that the friction coefficients of all specimens increase quickly and have a large fluctuation range at the initial stage of the friction test. Because this stage is the running-in stage of the friction wear process, and the contact between the friction pair and the tested specimen surface is unstable, the friction coefficient increases and has a large fluctuation range. The contact between both of them tends to remain steady as the wear duration increases and the above-mentioned phenomenon is mitigated. In this paper, the friction coefficients of each specimen at 20–30 min were selected as reference values and an average friction coefficient was calculated. The average friction coefficients and wear volumes of different specimens are in the following order from high to low: Substrate > S1 specimen > S3 specimen > S2 specimen > S4 specimen; see Figure 12 and Figure 13. Contrary to the microhardness trend, the average friction coefficient and wear volume of the coating are gradually reduced, which follows the Archard equation [38]. When the substrate is heated to 350 °C, the average friction coefficient and wear volume of the coating reach minimum values and are 0.54 and 3 × 10^−4^ cm^3^, respectively. This indicates that the wear property of the S4 specimen is excellent. The reason for this phenomenon is that it is mainly attributed to the formation of hard phases such as WC, M_7_C_3_, and M_23_C_6_ in the S4 cladding layer, which enhances the hardness of the coating. Therefore, the above phenomenon occurred. In addition, a comparison of the average friction coefficient and wear volume among the substrate, the S1 specimen, and the S4 specimen shows that (1) the average friction coefficient of the S1 specimen is 1.37 times that of S4, and the wear volume of the S4 specimens is 0.44 times that of S1; (2) the average friction coefficient of the S4 specimens is 0.70 times that of the substrate, and the wear volume of the S4 specimens is 0.24 times that of the substrate. To sum up, the temperature at which the substrate is preheated has a significant influence on the composite coating’s wear properties.

**Figure 14:**
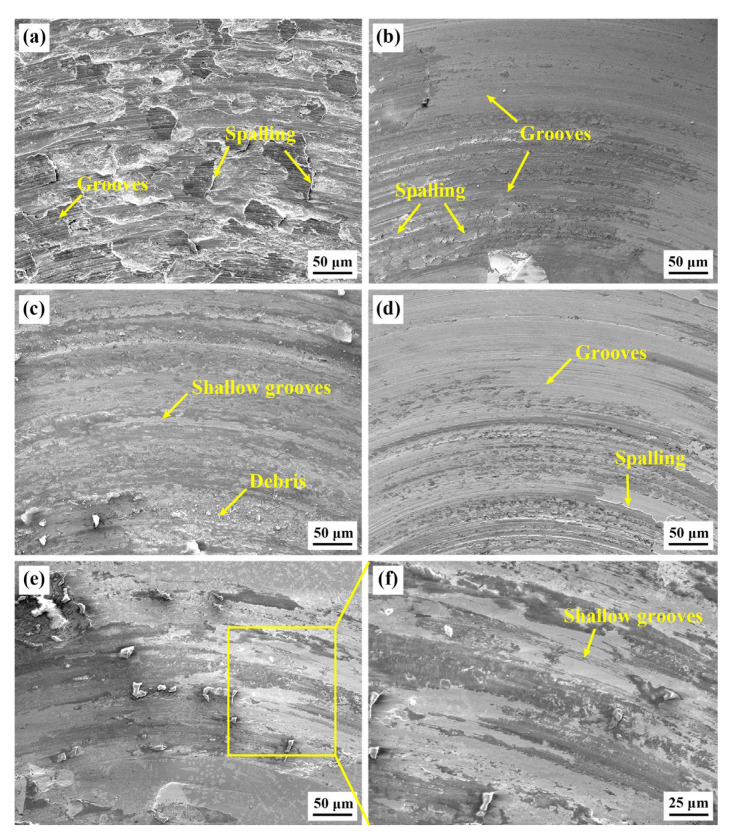
SEM wear morphologies of the Stellite 6/WC composite coatings of different specimens: (**a**) substrate; (**b**) S1; (**c**) S2; (**d**) S3; (**e**) S4; (**f**) magnification of the yellow rectangular area.

The SEM wear morphologies of the Stellite 6/WC composite coatings of different specimens are shown in Figure 14. It can be seen that the substrate’s wear morphologies in Figure 14a are the roughest among all coatings and there are many grooves and spalling pits in the surface, which shows that the substrate’s wear characteristic is a combination of adhesive wear and abrasive wear. This is because the 60Si2Mn substrate is much softer than the corresponding silicon nitride ceramic (Si_3_N_4_) ball friction pair. In the friction process, the grinding ball can easily break the substrate’s sliding surface under the effect of a normal load and generate shear stress on the substrate, causing many grooves and spalling pits in the specimen surface. Furthermore, as shown in Figure 14b,d, the above-mentioned phenomenon also exists in the wear surfaces of the S1 and S3 specimens, which shows that the wear characteristic of the S1 and S3 specimens is the combination of adhesive wear and abrasive wear. Figure 14c,e,f show the wear surfaces of the S2 and S3 specimens. When the substrate preheating temperature is 150 °C and 350 °C, the sliding surface of the Stellite 6/WC composite coating is smooth and has only shallow grooves and a small amount of debris, which shows that the wear characteristic of the specimens is slight abrasive wear. This indicates that preheating the substrate properly can play a role of self-lubrication, effectively improving the wear property of the coating.

## 4. Conclusions

In this paper, laser surface modification was performed on 60Si2Mn steel by the combination method of LC and substrate preheating, and the effect of different substrate preheating temperatures on the morphology, microstructure, and wear property of the cobalt-based tungsten carbide composite coating was comparatively investigated. The experimental results show that:

1. The surface height difference of the cobalt-based tungsten carbide composite coating falls initially and subsequently increases when the substrate preheating temperature rises, the coating height rises to 2.41 mm from 0.49 mm, and the cladding angle reduces dramatically.
2. By comparing four groups of coatings, the microstructure of the upper and middle parts of the cladding layer is mainly isometric crystal, and the microstructure of the lower part is columnar or cellular crystal at substrate preheating temperatures of room temperature, 150 °C, and 250 °C. At a substrate preheating temperature of 350 °C, the main structure of the cladding layer is various blocky, petaloid, and flower-like precipitates at a substrate preheating temperature of 350 °C.
3. The cobalt-based tungsten carbide composites are superior to 60Si2Mn steel in terms of hardness and wear properties. The hardness and wear resistance properties of the coating are optimal at a substrate preheating temperature of 350 °C. The hardness of the coating is 2.45 times higher than that of the substrate, and the average friction coefficient and the wear volume of the coating are 0.7 times and 0.24 times lower than those of the substrate, respectively.

## Figures and Tables

**Table 1:** Chemical composition (wt%) of 60Si2Mn steel substrate material.

Steel Designation	Nominal Composition, wt%						
	C	Si	Mn	Ni	Cu	Cr	Fe
60Si2Mn	0.56–0.6	1.5–2.0	0.6–0.9	≤0.35	≤0.25	≤0.35	Bal

**Table 2:** Chemical composition (wt%) of Stellite 6 cladding material.

Stellite Alloy Grade	Nominal Composition, wt%								
	C	Cr	Si	W	Fe	Mo	Ni	Co	Mn
Stellite 6	1.15	29.00	1.10	4.00	3.00	1.00	3.00	Bal	0.50

**Table 3:** Parameters of LC process.

Specimen	Laser Power [W]	Scan Speed [mm/s]	Feeding Rate [g/min]	Shielding Gas [L/min]	Preheating Temperature [°C]
S1	1500 W	5	13	10	Room temperature
S2					150
S3					250
S4					350

## Data Availability

Not applicable.
